# Neuroprotective Effects of Erucin against 6-Hydroxydopamine-Induced Oxidative Damage in a Dopaminergic-like Neuroblastoma Cell Line

**DOI:** 10.3390/ijms130910899

**Published:** 2012-08-30

**Authors:** Andrea Tarozzi, Fabiana Morroni, Cecilia Bolondi, Giulia Sita, Patrizia Hrelia, Alice Djemil, Giorgio Cantelli-Forti

**Affiliations:** Department of Pharmacology, Alma Mater Studiorum-University of Bologna, via Irnerio 48, 40126 Bologna, Italy; E-Mails: fabiana.morroni@unibo.it (F.M.); cecilia.bolondi2@unibo.it (C.B.); giulia.sita2@unibo.it (G.S.); patrizia.hrelia@unibo.it (P.H.); alice.djemil@studio.unibo.it (A.D.); giorgio.cantelliforti@unibo.it (G.C.-F.)

**Keywords:** Parkinson’s disease, 6-hydroxydopamine, dopaminergic cells, oxidative stress, neuronal death, erucin, glutathione, antioxidant activity, neuroprotection

## Abstract

Oxidative stress (OS) contributes to the cascade leading to the dysfunction or death of dopaminergic neurons during Parkinson’s disease (PD). A strategy to prevent the OS of dopaminergic neurons may be the use of phytochemicals as inducers of endogenous antioxidants and phase 2 enzymes. In this study, we demonstrated that treatment of the dopaminergic-like neuroblastoma SH-SY5Y cell line with isothiocyanate erucin (ER), a compound of cruciferous vegetables, resulted in significant increases of both total glutathione (GSH) levels and total antioxidant capacity at the cytosolic level. The increase of GSH levels was associated with an increase in the resistance of SH-SY5Y cells to neuronal death, in terms of apoptosis, induced by 6-hydroxydopamine (6-OHDA). The pretreatment of SH-SY5Y cells with ER was also shown to prevent the redox status impairment, in terms of intracellular ROS and O_2_^•−^ formation, and loss of mitochondrial membrane potential, early events that are initiators of the apoptotic process, induced by 6-OHDA. Last, the antiapoptotic and antioxidant effects of ER were abolished by buthionine sulfoximine, supporting the main role of GSH in the neuroprotective effects recorded by ER. These results suggest that ER may prevent the oxidative damage induced by 6-OHDA.

## 1. Introduction

The production and elimination of reactive oxygen species (ROS)—called oxidative stress (OS)—contributes to the cascade leading to the dysfunction or death of dopaminergic neurons in the substantia nigra (SN) during Parkinson’s disease (PD) [1–3]. In particular, the high levels of dopamine (DA) in the SN pathway are presumed to be an essential determinant for the vulnerability of dopaminergic neurons to OS. The catabolism of DA includes auto-oxidation into electrophilic DA quinone and ROS, such as superoxide anion (O_2_^•−^), and hydrogen peroxide (H_2_O_2_) [4]. Further, H_2_O_2_ generated by monoamine oxidase can react with free iron to produce hydroxyl radical (OH^•^), via the Fenton reaction, which increases the OS in dopaminergic neurons [5].

Among antioxidant endogenous molecules and enzymes, glutathione (GSH) provides the major line of defense for the protection of cells from oxidative and other forms of toxic stress [6]. Recent studies show that early depletion of glutathione (GSH) recorded in the SN appears to be responsible for subsequent OS, mitochondrial dysfunction, and dopaminergic neuron death in patients with PD [7]. Therefore, an effective neuroprotective strategy against OS in dopaminergic neurons could be to reinforce the cellular defense by using phytochemical inducers of antioxidant endogenous molecules.

Among phytochemicals, isothiocyanates (ITCs), derived from the glucosinolate hydrolysis found in abundance in cruciferous vegetables, have recently gained attention as potential neuroprotective compounds that induce antioxidant phase 2 enzymes and molecules through transcription factor Nrf2–dependent antioxidant response element activation [8]. In particular, aliphatic ITC sulforaphane (isothiocyanato-4-(methylsulfinyl)-butane or isothiocyanate SF), produced by glucoraphanin hydrolysis, the primary glucosinolate present in broccoli (*Brassica oleracea* L. ssp. *italica*), showed an ability to enhance the GSH, GSH-*S*-transferase (GST) and quinone reductase (QR) levels as well as detoxifying activity in both neurons and glial cells, including astroglial and microglial cells [9–11]. The closely related aliphatic ITC erucin (isothiocyanato-4-(methylthio)-butane or isothiocyanate erucin (ER)), derived from glucoerucin, a glucosinolate predominant in rocket salads (*Eruca sativa* Mill.), has also received attention due to its similar structure and *in vivo* inter conversion with SF [12]. In contrast to SF, the biological effects of ER are poorly supported by experimental data. Recent studies show the ability of ER to induce detoxification endogenous molecules in various animal and human tissues [13,14]. However, the potential neuroprotective effects of ER still remain unanswered.

In this study, we evaluated the ability of ER to improve total GSH levels and total antioxidant capacity (TAC) of a dopaminergic-like neuroblastoma SH-SY5Y cell line. In similar experimental conditions, we also assessed the neuroprotective effects of ER against 6-hydroxydopamine (6-OHDA)-induced oxidative damage in terms of impairment in intracellular redox state, loss of mitochondrial membrane potential (MMP) and cellular death. Similar to DA, 6-OHDA is an oxidant compound that mimics the oxidative stress and dopaminergic damage underlying PD via the same ROS, such as H_2_O_2_, O_2_^•−^ and OH^−^ [15,16].

## 2. Results and Discussion

**Figure 1:**
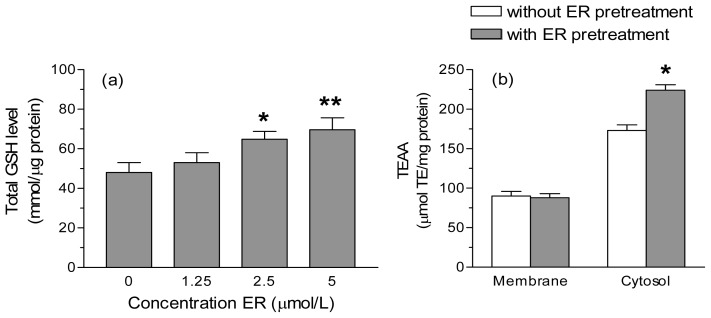
Isothiocyanate erucin (ER) enhances the total glutathione (GSH) level and the total antioxidant capacity (TAC) of SH-SY5Y cells. (**a**) SH-SY5Y cells were incubated with various concentrations of ER for 24 h. At the end of the incubation, GSH levels were measured as described in the Experimental Section. The values are shown as mean ± SEM of four independent experiments. * *p* < 0.05, ** *p* < 0.01 *versus* untreated cells at ANOVA with Dunnett *post hoc* test; (**b**) SH-SY5Y cells were treated with 5 μmol/L of ER for 24 h and cytosolic and membrane fractions were then separated as described in the Experimental Section. The cellular fractions were submitted to the ABTS^•+^ decolorization assay and the TAC of the fractions was expressed as μmol of trolox equivalent (TE) Antioxidant Activity per mg of protein (TEAA μmol/mg protein). The values are shown as mean ± SEM of three independent experiments. * *p* < 0.05 *versus* untreated cells at Student’s *t*-test.

Preliminary experiments showed that the treatment of SH-SY5Y cells with ER concentrations up to 5 μmol/L for 24 h did not affect intracellular redox state, MMP, cell growth and viability (data not shown). We first determined the ability of ER (1.25–5 μmol/L) to increase the total GSH levels in SH-SY5Y cells. Treatment of SH-SY5Y cells with 2.5 and 5 μmol/L of ER for 24 h resulted in significant increases (~35% and ~45%, respectively) in cellular GSH content (Figure 1a). We then evaluated whether the higher levels of GSH induced by ER effectively improves the TAC of SH-SY5Y cells at different subcellular levels, such as the membrane and cytosol. TAC was measured for its ability to quench the 2,2′-azinobis-(3-ethylbenzothiazoline-6-sulfonic acid (ABTS) radical cation (ABTS^•+^) and the results are expressed as trolox equivalent (TE) μmol/mg protein. The cytosolic, but not the membrane, fractions obtained from SH-SY5Y cells treated with 5 μmol/L of ER for 24 h showed significant increases of TAC compared to untreated cells (Figure 1b).

Taken together, these results show for the first time the ability of ER to induce GSH in SH-SY5Y cells. A similar induction of GSH together with other phase 2 enzymes, such as GST and QR, by ER was observed in human and rat non-neuronal tissues [13,14]. However, our results also suggest that ER as well as SF and other ITCs from cruciferous vegetables have particular induction patterns of cytoprotective proteins at neuronal level. Several *in vitro* and *in vivo* studies have shown that, through neurohormesis mechanisms, SF could activate Nrf2, a critical transcriptional activator for antioxidant and phase 2 genes [8,17,18]. In this context, other studies with non-neuronal tissues, ER and SF behaved in a similar way, indicating that the oxidation state of sulfur has little impact on the ability of these aliphatic ITCs to increase the total GSH and QR [13]. Although we did not measure Nrf2 levels, it is reasonable to suppose that it may contribute to GSH enhancement mediated by ER in neuronal cells.

**Figure 2:**
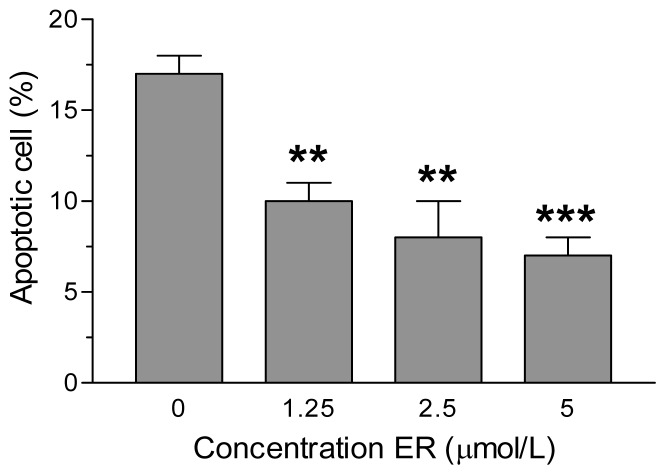
ER prevents 6-OHDA-induced apoptosis in SH-SY5Y cells. SH-SY5Y cells were incubated with various concentrations of ER for 24 h and then treated with 6-OHDA (200 μmol/L) for 2 h at 37 °C. After a further 15 h of incubation in medium without 6-OHDA, the apoptotic cells were determined by Annexin V staining and fluorescence microscopy as described in the Experimental Section. The values are expressed as a percentage of total number of apoptotic cells and shown as mean ± SEM of three/four independent experiments. ** *p* < 0.01, *** *p* < 0.001 *versus* untreated cells at ANOVA with Dunnett *post hoc* test.

To ascertain whether the increases of the total GSH level and TAC recorded in SH-SY5Y cells could really translate into neuroprotective effects, we subsequently evaluated the ability of the SH-SY5Y cells under identical experimental conditions to prevent the increase of neuronal death, in terms of apoptosis, induced by 6-OHDA. As reported in Figure 2, the pre-treatment of SH-SY5Y cells for 24 h with ER led to a strong dose-dependent decrease of apoptotic cells elicited by 6-OHDA. The observed decrease of apoptosis was significant for all ER concentrations employed (1.25–5 μmol/L) and the maximum inhibition was ~59% with respect to cells treated with 6-OHDA alone. Further, the degree of apoptosis inhibition shown by pre-treatment of SH-SY5Y cells with similar concentrations of ER against 6-OHDA toxicity significantly correlated with elevation of total GSH levels (*r* = 0.8146, *p* < 0.05). In order to evaluate the role of GSH in ER neuroprotection, we used buthionine sulfoximine (BSO), which irreversibly inhibits gamma-glutamylcysteine synthetase, the first enzyme in the GSH biosynthesis pathway. The addition of BSO 400 μmol/L to treatment of SH-SY5Y cells with ER 5 μmol/L for 24 h abolished the antiapoptotic effects observed with ER alone against toxicity induced by 6-OHDA (sham: 3% ± 1%; BSO: 4% ± 1%; 6-OHDA: 16% ± 3%; 6-OHDA + ER: 7% ± 4%; 6-OHDA + ER + BSO: 13% ± 4%; results are expressed as percentage of apoptotic cells).

These findings suggest that total GSH could play an important contribution to the neuroprotective effects of ER in SH-SY5Y cells. However, it is relevant to point out that at concentrations <2.5 μM ER can contribute to attenuate the apoptosis elicited by 6-OHDA in absence of significant increase of GSH level. It is pertinent to emphasize that antiapoptotic effects of ER could also be ascribed its ability to active subsidiary neuronal survival mechanisms. Recent studies indicate that PI3K/Akt and MEK/ERK signalling pathways are important intracellular mediators in ER-induced phase II enzymes in intestinal epithelial Caco-2 cells [19]. Among these intracellular signaling pathways, the ability of PI3K/Akt to affect 6-OHDA-induced neuronal death in SH-SY5Y cells has been suggest [20,21]. Whether the PI3K/Akt and other cell survival kinases are also involved in ER’s neuroprotective effects warrants further investigation.

**Figure 3:**
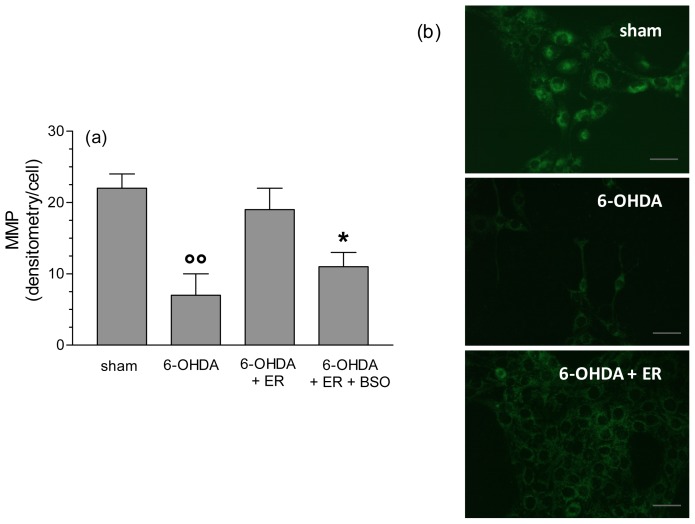
ER prevents the loss of mitochondrial membrane potential (MMP) induced by 6-OHDA in SH-SY5Y cells. (**a**) SH-SY5Y cells were incubated with ER (5 μmol/L) in absence or presence of buthionine sulfoximine (BSO) (400 μmol/L) for 24 h and then treated with 6-OHDA (200 μmol/L) for 2 h. At the end of incubation, MMP was determined using a fluorescence probe, rhodamine 123 (Rh-123), as described in the Experimental Section. Four randomly selected areas with 50–100 cells in each were analyzed under a fluorescence microscope and the values obtained are expressed as densitometry/cell. Values are shown as mean ± SEM of four independent experiments: ∘∘ *p* < 0.01 *versus* untreated cells, * *p* < 0.05 *versus* cells treated with 6-OHDA + ER at ANOVA with Bonferroni *post hoc* test; (**b**) representative images of MMP. Scale bars: 100 μm.

**Figure 4:**
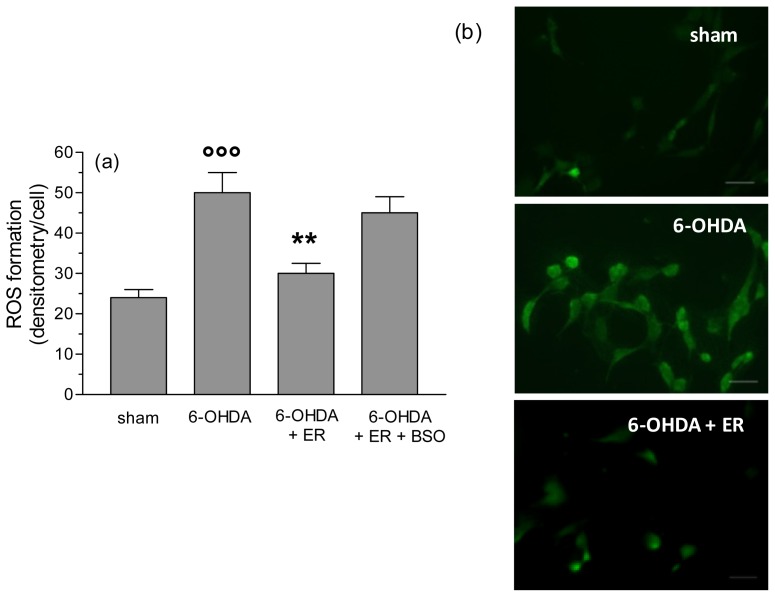
ER counteracts 6-OHDA-induced intracellular ROS formation in SH-SY5Y cells. (**a**) SH-SY5Y cells were incubated with ER (5 μmol/L) in absence or presence of BSO (400 μmol/L) for 24 h and then treated with 6-OHDA (200 μmol/L) for 2 h. At the end of incubation, ROS formation was determined using a fluorescence probe, DCFH-DA, as described in the Experimental Section. Four randomly selected areas with 50–100 cells in each were analyzed under a fluorescence microscope and the values obtained are expressed as densitometry/cell. Values are shown as mean ± SEM of four independent experiments: ∘∘∘ *p* < 0.001 *versus* untreated cells, ** *p* < 0.01 *versus* cells treated with 6-OHDA at ANOVA with Bonferroni *post hoc* test; (**b**) representative images of ROS formation. Scale bars: 100 μm.

**Figure 5:**
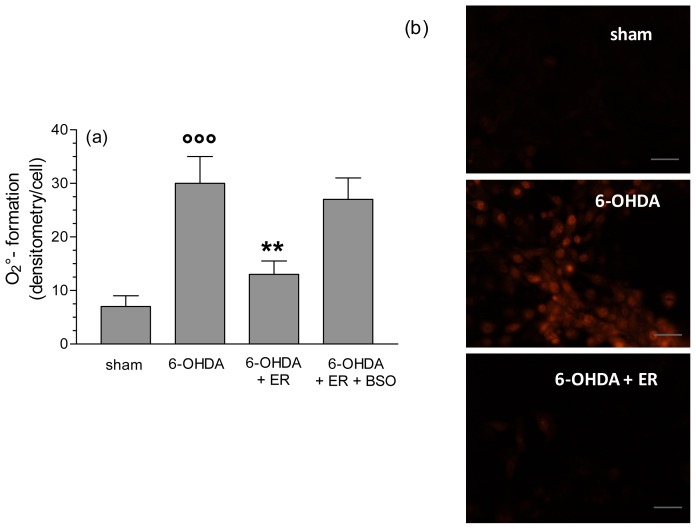
ER counteracts 6-OHDA-induced intracellular O_2_^•−^ formation in SH-SY5Y cells. (**a**) SH-SY5Y cells were incubated with ER (5 μmol/L) in absence or presence of BSO (400 μmol/L) for 24 h and then treated with 6-OHDA (200 μmol/L) for 2 h. At the end of incubation, O_2_^−−^ formation was determined using a fluorescence probe, DHE, as described in the Experimental Section. Four randomly selected areas with 50–100 cells in each were analyzed under a fluorescence microscope and the values obtained are expressed as densitometry/cell. Values are shown as mean ± SEM of four independent experiments: ∘∘∘ *p* < 0.001 *versus* untreated cells, ** *p* < 0.01 *versus* cells treated with 6-OHDA at ANOVA with Bonferroni *post hoc* test; (**b**) representative images of O_2_^•−^ formation. Scale bars: 100 μm.

To clarify the antioxidant and protective mechanisms underlying the antiapoptotic effects of ER, we also evaluated its ability to prevent the redox status impairment, in terms of MMP loss, and intracellular ROS and O_2_^•−^ formation, early events that are initiators of the apoptotic process, induced by 6-OHDA. As reported in Figure 3, pre-treatment of SH-SY5Y cells with ER (5 μmol/L) for 24 h showed a significant inhibition of MMP loss elicited by 6-OHDA. Further, the same treatment with ER 5 μmol/L also showed a strong inhibitory effect on 6-OHDA-induced total ROS and O_2_^•−^ formation in SH-SY5Y cells using fluorescent probe 2′,7′-dichlorodihydroflurescein diacetate (DCFH-DA) and dihydroethidium (DHE), respectively (Figures 4 and 5). Remarkably, this finding was abolished by adding BSO (400 μmol/L) to the treatment with ER and 6-OHDA, supporting the predominant role of GSH in the indirect antioxidant effects displayed by ER (Figures 3–5).

Among our findings, it emerged that elevation of GSH in SH-SY5Y cells after treatment with ER could be a determinant to prevent the early redox status impairment and loss of MMP responsible for apoptosis. It is noted that ROS generated by dopamine or through its 6-OHDA metabolite are able to induce oxidative damage to the mitochondria, which is a critical intracellular target for the pathogenesis of PD [22]. Recent studies suggest that the free radical acting on the components of the electron transport system and uncoupling mitochondrial respiration is the OH^•^ generated from O_2_^•−^ [16,23]. In this context, it is plausible that the GSH induced by ER reacts directly with O_2_^•−^ preventing the formation of OH^•^, an ROS with a non-short half-life, which is involved in both mitochondrial impairment and cellular death. Further, the increased amount of GSH in cytoplasm by ITC inducers including ER may also lead to a concurrent elevation of GSH in the mitochondrial compartment avoiding the thiol oxidation of the complex I, an event responsible for mitochondrial dysfunction [24].

## 3. Experimental Section

### 3.1. Reagents

ER was obtained from LKT Laboratories (St. Paul, MN, USA) and dissolved in dimethyl sulfoxide (DMSO) to a stock solution of 5 mmol/L. For experiments, this stock was then diluted with Dulbecco’s modified Eagle’s medium (DMEM). The concentration of DMSO in all controls and treatments did not exceed 0.1% (*v*/*v*). ABTS, DCFH-DA, DHE, 5,5′-dithiobis 2-nitrobenzoic acid (DTNB), 6-OHDA and Rh-123 were obtained from Sigma-Aldrich (St. Louis, MO, USA). The Annexin-V-FLUOS Staining Kit was obtained from Roche Diagnostics (Mannheim, Germany). All other reagents were of the highest grade of purity commercially available.

### 3.2. Cell Culture and Treatments

SH-SY5Y cell line was routinely grown at 37 °C in a humidified incubator with 5% CO_2_ in DMEM supplemented with 10% fetal bovine serum (FBS), 2 mmol/L glutamine, 50 U/mL penicillin and 50 μg/mL streptomycin. To evaluate GSH levels and neuronal death, in terms of apoptosis, the SH-SY5Y cells were seeded in cultures dishes (size 100 mm) at 4 × 10^6^ and 1.5 × 10^6^ cells/dish, respectively. To assess intracellular ROS and O_2_^•−^ formation, and mitochondrial membrane potential, the SH-SY5Y cells were cultured in BD Falcon™ 8–well Culture slides (surface area 0.7 cm^2^/well) at 1 × 10^4^ cells/well. All experiments were performed after 24 h of incubation at 37 °C in 5% CO_2_.

To determine the ability of ER to modulate the GSH levels, SH-SY5Y cells were incubated for 24 h, with ER in a concentration range of 1.25–5 μmol/L at 37 °C in 5% CO_2_. To estimate the neuroprotective effects of ER, the SH-SY5Y cells incubated with ER as reported above were then treated with 6-OHDA (200 μmol/L) for 2 h. At the end of incubation, the MMP, intracellular ROS and O_2_^•−^ formation were determined, while after a further 15 h of incubation without 6-OHDA, the apoptosis was determined.

### 3.3. Determination of GSH Levels

Cellular GSH levels were determined by the glutathione reductase-coupled DTNB assay in 96-well plates. Briefly, SH-SY5Y cells were washed with cold phosphate buffered saline (PBS). Cells were subsequently scraped and collected in 1 mL of PBS and centrifuged for 10 min at 7000*g* at 4 °C, after which the pellet was lysed with 500 μL of 0.1% Triton X-100. Cells were then homogenized and allowed to stand at 4 °C for 5 min. Cells were centrifuged at 11,000*g* for 15 min at 4 °C. Then, 25 μL of supernatant were transferred into a 96-well plate and 25 μL of cold sulfosalicylic acid (5%) were added to each well. The plate was shaken for 2 min and 125 μL of the reaction buffer (containing the DTNB) were added. The plate was shaken for 15 s and the optical density was measured at 415 nm using a microplate reader (Spectra model Classic, TECAN^®^, Männedorf, Switzerland). The concentration of GSH was then calculated using a standard calibration curve and expressed as millimoles per microgram of total protein per assay.

### 3.4. Determination of TAC in Membrane and Cytosolic Fractions

TAC, a marker of cellular antioxidant status, was measured on the cytosolic and membrane enriched fractions of SH-SY5Y cells as we previously reported [25]. This method is based on the ability of the exogenous and endogenous antioxidant molecules in the cell extract to reduce ABTS^•+^. Briefly, SH-SY5Y cells were washed 3 times with cold PBS. Cells were subsequently collected in 1 mL of PBS and centrifuged for 10 min at 7000*g* at 4 °C, after which the supernatant was removed and the cells were washed with 1 mL of PBS. This was repeated a further 2 times, and the pellet was finally reconstituted in 600 μL of 0.05% Triton X-100. Cells were then homogenized and allowed to stand at 4 °C for 30 min. Cytosolic and membrane enriched fractions were subsequently separated by centrifugation at 11,000*g* for 15 min at 4 °C. Membrane and cytosolic fractions were stored at −20 °C.

TAC in cell fractions was then determined by the decoloration of ABTS^•+^, in terms of quenching of absorbance at 740 nm. Values obtained for each cellular fraction sample were compared with the concentration-response curve of a standard Trolox solution, and expressed as micromolar of TE Antioxidant Activity per mg of protein.

### 3.5. Determination of Neuronal Apoptosis

Apoptosis in terms of membrane phosphatidylserine exposure was evaluated using the Annexin-V-FLUOS Staining Kit, according to the manufacturer’s instructions. Briefly, SH-SY5Y cells were scraped, suspended at 2.5 × 10^5^/mL, and washed with PBS. The cells were incubated with 100 μL of Annexin-V-FLUOS labeling solution at room temperature in the dark for 15 min. To determine the percentage of stained cells, four randomLy selected areas with 50–100 cells in each were examined under a fluorescence microscope (Zeiss Axio Imager M1, Oberkochen, Germany) at λ_excitation_ = 488 nm and λ_emission_ = 518 nm. The values are expressed as percentage of apoptotic cells and calculated by the formula:

The value=(annexin V-positive cells/n total cells)×100

### 3.6. Determination of MMP

MMP was determined using the fluorescent probe, Rh-123 (λ_excitation_ = 485 nm, λ_emission_ = 535 nm). Briefly, SH-SY5Y cells were washed and incubated with Rh-123 (5 μmol/L) for 30 min in the dark. After removal of the probe and further washing with PBS, MMP was measured under a fluorescence microscope (Zeiss Axio Imager M1). Fluorescence images were captured with an AxioVision image recording system computer. Four randomly selected areas with 50–100 cells in each were analyzed and the values obtained are expressed as densitometry/cell.

### 3.7. Determination of Intracellular ROS and O_2_^•−^ Formation

ROS and O_2_^•−^ formation was determined using the fluorescent probe, DCFH-DA (λ_excitation_ = 485 nm, (λ_emission_ = 535 nm) and DHE ((λ_excitation_ = 380 nm, (λ_emission_ = 445 nm), respectively. Briefly, SH-SY5Y cells were washed and incubated with DCFH-DA (5 μmol/L) or DHE (10 μmol/L) for 30 min in the dark. After removal of the probe, cells were washed with PBS and incubated with DMEM serum free for 1 h at 37 °C. Intracellular ROS and O_2_^•−^ formation was measured under a fluorescence microscope (Zeiss Axio Imager M1). Fluorescence images were captured with an AxioVision image recording system computer. Four randomly selected areas with 50–100 cells in each were analyzed and the values obtained are expressed as densitometry/cell.

### 3.8. Statistical Analysis

Data are reported as mean ± SEM of at least 3 independent experiments. Statistical analysis was performed using one-way ANOVA with Dunnett or Bonferroni *post hoc* test and Student’s *t*-test, as appropriate. Pearson’s test was also used to assess correlation. Differences were considered significant at *p* < 0.05. Analyses were performed using GraphPad PRISM software (version 3.0; GraphPad Software, Inc.: La Jolla, CA, USA, 2002) on a Windows platform.

## 4. Conclusions

ER shows an interesting profile of antioxidant and neuroprotective effects similar to those observed with related ITC SF. These findings suggest that the neuroprotective effects of SF recorded in various neurodegeneration animal models could also be ascribed to its metabolic conversion to ER [26,27]. Moreover, cruciferous vegetables, including rocket salads, that are rich in glucoerucin, the precursor of ER, may represent potential sources of phytochemicals with neuroprotective properties that may play an important role in preventing PD. In this context, further clinical studies are warranted to investigate the potential *in vivo* neuroprotective role of ER.
